# Association of PET-measured myocardial flow reserve with echocardiography-estimated pulmonary artery systolic pressure in patients with hypertrophic cardiomyopathy

**DOI:** 10.1371/journal.pone.0212573

**Published:** 2019-03-20

**Authors:** Min Zhao, Min Liu, Jeffrey P. Leal, Benjamin M. W. Tsui, Dean F. Wong, Martin G. Pomper, Yun Zhou

**Affiliations:** 1 Department of Nuclear Medicine, Xiangya Hospital, Central South University, Changsha, China; 2 The Russell H. Morgan Department of Radiology and Radiological Science, Johns Hopkins School of Medicine, Baltimore, Maryland, United States of America; 3 Mallinckrodt Institute of Radiology, Washington University in St. Louis School of Medicine, St. Louis, Missouri, United States of America; Scuola Superiore Sant'Anna, ITALY

## Abstract

**Background:**

Pulmonary hypertension (PH) is a known complication of HCM and is a strong predictor of mortality. We aim to investigate the relationship between microvascular dysfunction measured by quantitative PET and PH in HCM patients.

**Methods:**

Eighty-nine symptomatic HCM patients were included in the study. Each patient underwent two 20-min ^13^N-NH_3_ dynamic PET scans for rest and stress conditions, respectively. A 2-tissue irreversible compartmental model was used to fit the segments time activity curves for estimating segmental and global myocardial blood flow (MBF) and myocardial flow reserve (MFR). Echocardiographic derived PASP was utilized to estimate PH.

**Results:**

Patients were categorized into two groups across PASP: PH (PASP > 36 mmHg) and no-PH (PASP ≤ 36 mmHg). patients with PH had larger left atrium, ratio of higher inflow early diastole (E) and atrial contraction (A) waves, E/A, and ratio of inflow and peak early diastolic waves, E/e', significantly reduced global stress MBF (1.85 ± 0.52 vs. 2.13 ± 0.56 ml/min/g; *p* = 0.024) and MFR (2.21 ± 0.57 vs. 2.62 ± 0.75; *p* = 0.005), while the MBFs at rest between the two groups were similar. There were significant negative correlations between global stress MBF/MFR and PASP (stress MBF: r = -0.23, *p* = 0.03; MFR: r = -0.32, *p* = 0.002); for regional MBF and MFR measurements, the highest linear correlation coefficients were observed in the septal wall (stress MBF: r = -0.27, *p* = 0.01; MFR: r = -0.31, *p* = 0.003). Global MFR was identified to be independent predictor for PH in multivariate regression analysis.

**Conclusion:**

Echocardiography-derived PASP is negatively correlated with global MFR measured by ^13^N-NH_3_ dynamic PET. Global MFR is suggested to be an index of PH in HCM patients.

## Introduction

Hypertrophic cardiomyopathy (HCM) is the most common heritable cardiomyopathy, characterized by cardiac hypertrophy and phenotypic heterogeneity [1, 2]. Although much attention has focused on the left-sided pathophysiology, alterations in pulmonary hemodynamics may also be evident. Patients with HCM may develop to pulmonary hypertension (PH) due to elevated left-sided diastolic pressures, secondary to diastolic dysfunction, and in a minority of cases to the primary pulmonary vascular resistance [3]. A few studies have identified that PH in HCM is associated with a poor prognosis even with mild elevations in pulmonary pressures [3–5]. However, there is considerable variability in the clinical course of HCM patients, additional modalities which could identify patients at risk for PH and adverse outcomes might be useful in the clinical evaluation of HCM patients.

Coronary microvascular dysfunction is an central physiopathology in HCM, and has major prognostic implications as well [6]. Growing evidence suggests that the assessment of microvascular function detected by quantitative positron emission tomography (PET) could play an important role in the evaluation and management of myocardial ischemia in patients with HCM [7]. Accordingly, impaired hyperemic myocardial blood flow (MBF) and myocardial flow reserve (MFR) were regarded as equivalent to the microvascular dysfunction [8]. To date, it remains unknown whether microvascular function and PH are associated. In the present study, we used ^13^N-NH_3_ dynamic PET imaging to explore the possible quantitative relationship between MBF/MFR and pulmonary artery systolic pressures (PASP) measured by Doppler echocardiography.

## Materials and methods

### Study population

This project was reviewed and approved by the John Hopkins Institutional Review Board (No. 00029377), All procedures and methods were performed in accordance with the updated guidelines and regulations. All patients provided consent to use clinical data for research purposes, and written informed consents were obtained from all participants. The retrospective study enrolled 118 patients with HCM at Johns Hopkins Hospital, Baltimore, USA, from June 2011 to December 2015 referred to cardiac PET/CT. The clinical diagnosis of HCM was based on 2-dimesional echocardiographic evidence of LV hypertrophy (maximal wall thickness ≥15 mm) in the absence of other cardiac or systemic disease such as obstructive coronary artery disease (> 50% diameter stenosis) by invasive coronary angiography or computed tomography angiography, hypertension, sarcoidosis capable of producing hypertrophy [1, 2]. All patients underwent comprehensive echocardiographic evaluation and ^13^N-NH_3_ dynamic PET imaging within a 1-month period. 29 patients were excluded from this analysis because of moderate or severe valval heart disease, severe lung disease and unavailable measurement of peak tricuspid regurgitation gradients, as well as patients whose PET images were missing or uninterpretable owing to poor image quality.

### Echocardiography

Two-dimensional Doppler echocardiographic studies were performed using a GE Vivid 7 or Ezuochuang-9 ultrasound machine (GE Ultrasound, Milwaukee, WI) on each patient. Echocardiographic assessments were based on the current guideline [9]. Peak instantaneous LV outflow gradient was estimated with continuous wave Doppler at rest and after exercise to elicit latent obstruction. Rest obstruction was defined as gradient ≥ 30 mmHg at rest, latent obstruction as gradient < 30 mmHg at rest, but gradient ≥ 30 mmHg on provocation, and no obstruction as gradient < 30 mmHg at rest and on provocation. Inflow early diastole (E) and atrial contraction (A) waves were assessed for the E/A ratio. The peak early diastolic wave (e') was used to calculate the E/e´ ratio. The PASP was obtained by addition of estimated right atrial pressure based on inferior vena cava size and collapsibility and trans-tricuspid gradient calculated from the modified Bernoulli equation [4 times the velocity (in m/s) of tricuspid regurgitation jet (TRV) square] [10]. Pulmonary stenosis or right ventricular outfiow tract obstruction were excluded. PH was identified as PASP > 36 mmHg and classified as mild (PASP 37~50 mmHg) and moderate or severe (PASP> 50 mmHg) [4, 11].

### PET/CT acquisition

All patients underwent cardiac PET/CT scanning using a GE 64-slice Discovery Rx VCT PET/CT system (GE Healthcare, Waukesha, Wisconsin). Patients were positioned with the assistance of a computed tomographic (CT) scout, a low-dose CT scan (120 kv, 30 mA) was performed for attenuation correction of PET emission data. Subsequently, 20-min dynamic PET images were acquired using a same-day rest/stress protocol [12, 13] as follows: approximately 370 MBq ^13^N-NH_3_ was injected intravenously as a bolus (using a power injector as constant rate of 1200 ml/h), and a list-mode dynamic PET scan was obtained over 20 minutes. Approximately 60 minutes after injection of the rest dose, Regadenoson (Lexiscan, Gilead Sciences Inc., Foster City, California) (0.4 mg/5 ml) was administered for vasodilator stress, and the stress PET scan was started about 30 sec after Regadenoson administration. Heart rate, blood pressure, and a 12-lead electrocardiogram were recorded before, during, and after completion of the stress protocol.

The attenuation- and decay-corrected 36-frame (20×6, 5×12, 4×30, 5×60, 2×300 seconds) dynamic PET images (volume size:128×128×47, and voxel size: 3.27×3.27×3.27 mm in x, y, z direction) and gated PET images (8 bins per cardiac cycle, volume size:128×128×47, voxel size: 3.27×3.27×3.27 mm) were reconstructed using an iterative ordered-subset expectation-maximization (OS-EM) algorithm (2 iterations, 21 subsets) with post-processing filtering (Butterworth, order 0.5 cycles/cm).

### MBF quantification

All reconstructed dynamic PET images were transferred to a workstation for image processing and quantification using the PCARDP tool (PMOD Technologies, Zurich, Switzerland, version 3.4). The images were reoriented along the heart axis, and segmented into the AHA 17-segments within the detected endo- and epicardial borders [14]. A 2-tissue irreversible compartment model (2TCM) with four parameters (F, k_2_, k_3_, V_b_) (15) was employed for fitting the 17-segments tracer time-activity curve (TAC). Volume of interests (VOIs) were manually drawn in the mitral of LV and in the right ventricle on PET images. The LV TACs and the average of LV and RV TACs were used as input function and blood volume correction, respectively. Our in-house software were used for model fitting [15]. The septal, anterior, lateral, inferior and global flow (S1 Table) and MFR were calculated from the 17 segmental MBFs and MFRs. Coronary vascular resistance was calculated as the mean arterial blood pressure divided by MBF at rest (maximal coronary vascular resistance) and stress (minimal coronary vascular resistance) as follows:

CVR=0.33×((2×diastolicpressure)+systolicpressure)/MBF

### Gated PET evaluation

Stress and rest LV ejection fraction (LVEF), LV end-diastolic volumes (LVEDV) and LV end-systolic volumes (ESV) were automatically calculated from gated datasets by using QGS package (Cedars Sinai, Los Angeles, California). The LVEF reserve was computed as stress LVEF minus rest LVEF. A drop larger than– 5 LVEF units was considered abnormal LVEF reserve as previously reported [16].

### Statistical analysis

Simple statistics including mean, standard deviation (SD), and proportions were calculated for continuous variables and categorical variables, respectively. The comparison between groups of continuous samples was performed with a Student’s t test, Mann–Whitney U test and one-way ANOVA depending on the nature of data. Categorical variables between groups were compared using the χ^2^ test. Spearman’ correlation coefficients were calculated for potential correlation between MBF/MFR and other variables. Univariable and multivariable linear regression analyses were performed to study the independent contributions of various parameters on PASP. A *p* < 0.05 was required for statistical significance. The IBM SPSS 23.0 software (IBM Corp, Somers, NY) was used for all statistical analysis.

## Results

### Clinical and echocardiographic features

Overall, the PH (PASP > 36 mmHg) was observed in 31 (35%) patients. Moderate or severe PH (PASP > 50 mmHg) was presented in 5 (6%) patients. Clinical and echocardiographic features of the patients with PH versus patients without PH are summarized in Table 1. There is no significant difference of age, gender, BMI, cardiovascular risk factors and symptoms. Non-invasive parameters of diastolic function including E/A ratio and medial E/e’ ratio were significantly worse in the PH group versus no-PH group (*p* < 0.01; *p* < 0.05, respectively). In addition, PH group had more increased left atrial size (4.4 ± 0.9 vs. 4.0 ± 0.6 cm, *p*<0.05) than no-PH group.

**Table 1:** Clinical and echocardiographic findings of HCM patients with and without PH.

Characteristics	Total (n = 89)	No PH (n = 58)	PH (n = 31)	*p*-value
**Clinical data**				
Age, years	52±15	52±13	52±17	0.893
Sex, male, n(%)	45(51)	31(53)	14(45)	0.456
BMI, kg/m^2^	28.5±4.7	28.6±4.8	28.4±4.5	0.850
NYHA Class III/IV, n(%)	51(58)	32(56)	19(61)	0.640
Dyspnea, n(%)	64(73)	39(68)	25(81)	0.219
**Risk factors**				
Hypertension, n(%)	45(50)	29(50)	16(52)	0.885
Dyslipidemia, n(%)	48(54)	30(52)	18(58)	0.568
Diabetes mellitus, n(%)	12(13)	9(15)	3(10)	0.442
Smoking, n(%)	30(34)	20(34)	10(32)	0.832
**Medications**				
β-Blockers	65(73)	41(71)	24(77)	0.495
Calcium channel blockers	28(31)	19(33)	9(29)	0.718
Diuretics	16(18)	10(17)	6(19)	0.805
Disopyramide	3(3)	0(0)	3(10)	**0.016**
**Echocardiographic parameters**				
Maximal LV thickness, cm	2.0±0.5	2.0±0.4	2.1±0.5	0.128
Rest LVOT gradient, mmHg	29±29	27±28	33±32	0.359
Provoked LVOT gradient, mmHg	68±56	67±59	72±51	0.513
LVEF(%)	67±7	67±6	67±8	0.662
Moderate/severe MR, n(%)	16(19)	8(14)	8(27)	0.160
LAD, cm	4.1±0.7	4.0±0.6	4.4±0.9	**0.015**
E/A ratio	1.5±0.7	1.3±0.6	1.8±0.9	**0.004**
E/e’ ratio	18.3±8.7	16.9 ±7.1	21.5±10.5	**0.021**

Data are expressed as mean ± standard deviation or number of the patients(percentage).

PH:pulmonary hypertension; BMI: body mass index; NYHA: New York Heart Association; LVOT: left ventricular outflow tract; LVEF: left ventricular ejection fraction; MR: mitral regurgitation; LAD: left atrial diameter; E/A:ratio of peak early diastolic velocity(E)/peak atrial filling velocity(A); E/e’: ratio of peak early diastolic velocity (E) /peak early diastolic velocity of the septal mitral annulus (e’).

### Regional and global MBF

**Fig 1:**
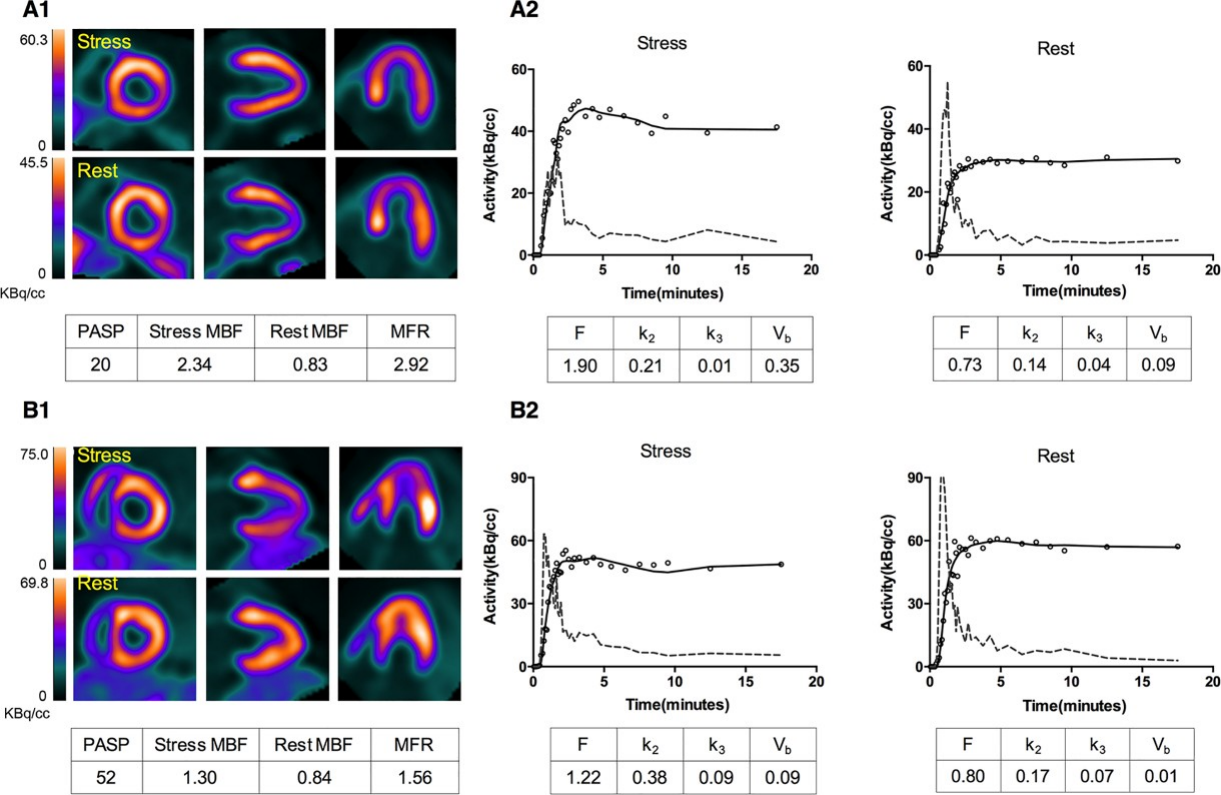
Representative cases of HCM with or without PH. (A1, B1): patient A is a 56-year-old female with normal PASP showing no evidence of vasodilator stress-induced myocardial ischemia; patient B is a 37-year-old female with elevated PASP revealing global myocardial ischemia but most severe in mid to apical regions of lateral and anterior walls; Lines from left to right: short axial slice; vertical axial slice; horizontal axial slice; (A2, B2): Time-activity curves at stress and rest. Dashed line: arterial blood; Hollow dots: myocardial time-activity curve of apical-lateral segment measured by PET; Solid line: myocardial time-activity curve predicted by the model.

**Fig 2:**
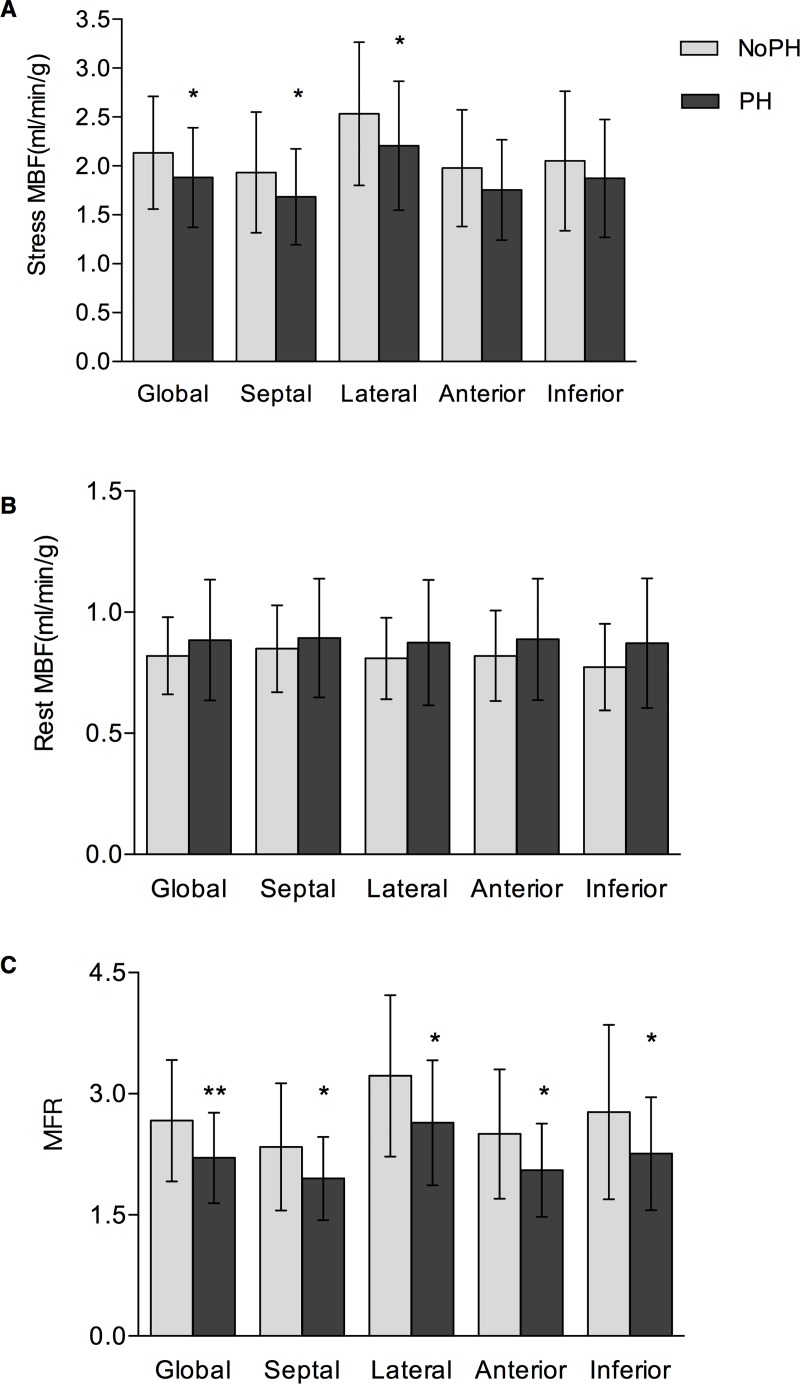
Comparison of global and regional MBF/ MFR between HCM patients with and without PH. A: stress MBF; B: rest MBF; C: MFR. *p<0.05, **p<0.01 for comparison between PH versus no PH.

The last 18-min mean stress/rest PET images with representative global MBF and MFRs for HCM patients with and without PH are demonstrated by Fig 1A-1 and 1B-1. The apical-lateral kinetic modeling results for the two typical HCM patients are illustrated by Fig 1A-2 and 1B-2. The model parameters of F, k_2_, k_3_ and V_b_ estimated from 17-segmental TACs based kinetic modeling for all patients are summarized in the S1 Table. Simple statistics of global MBF and MFR estimates for PH and no-PH HCM patients are included in Table 2. Patients with PH had evidence of significantly lower global stress MBF (1.85 ± 0.52 vs. 2.13 ± 0.56 ml/min/g; p < 0.05) and MFR (2.21 ± 0.57 vs. 2.62 ± 0.75; p < 0.01), while a higher global minimal CVR (52.31 ± 13.35 vs.44.18 ± 12.51 ml/min/g/mmHg; p < 0.01). Similarly, there were much more patients being classified as having abnormal stress MBF or MFR in group of PH than those patients in group of no-PH (*p* < 0.05 and *p* < 0.01 for MBF ≤ 1.8 ml/min/g and MFR ≤ 2.5, respectively. S2 Table). On the other hand, when compared the regional parameters between these two groups, septal and lateral MBFs at stress, as well as each regional MFR were significantly depressed in PH patients (Fig 2).

**Table 2:** Global PET parameters of HCM patients with and without PH.

Characteristics	Total (n = 89)	No PH (n = 58)	PH (n = 31)	*p*-value
SBP, mmHg	130±19	128±20	135±18	0.103
DBP, mmHg	75±12	74±12	78±11	0.145
Global stress MBF, ml/min/g	2.03±0.56	2.13±0.56	1.85±0.52	**0.012**
Global rest MBF, ml/min/g	0.85±0.20	0.84±0.17	0.86±0.24	0.952
Global MFR, unitless	2.48±0.71	2.62±0.75	2.21±0.57	**0.005**
Maximal CVR, ml/min/g/mmHg	110.78±30.27	110.74±26.35	110.87±37.09	0.586
Minimal CVR, ml/min/g /mmHg	46.98±13.31	44.18±12.51	52.31±13.35	**0.001**

Data are expressed as mean ± standard deviation.

PH:pulmonary hypertension; SBP: systolic blood pressure; DBP: diastolic blood pressure; MBF: myocardial blood flow; MFR: myocardial flow reserve; CVR: coronary vascular resistance.

### PET-derived LVEF

At rest, PET-derived LVEF was similar between the two groups (*p* > 0.05). During vasodilator stress, patients with PH exhibited impaired LVEF compared to the patients without PH (46 ± 12 vs. 52 ± 12%; *p* < 0.05). As a consequence, group of PH yielded lower LVEF reserve values (-8 ± 6 vs. -5 ± 5%; *p* < 0.01) and high prevalence of abnormal LVEF reserve (64% vs. 40%; *p* < 0.05) as above defined (S3 Table).

### LVOT obstruction

In the study, 32 (36%) patients were classified as no-obstructive HCM, 32 (36%) had latent obstruction and 25 (28%) had rest obstruction. Obstructive HCM patients were more likely to have higher E/e’ ratio; Otherwise, there was no significant difference in stress MBF, MFR, minimal CVR and PASP among the 3 groups, as depicted in Table 3.

**Table 3:** Characteristics of HCM patients with and without obstruction.

Characteristics	Non-obstructive HCM (n = 32)	Latent obstructive HCM (n = 32)	Obstructive HCM (n = 25)	*p*-value
Global stress MBF, ml/min/g	2.01±0.58	2.11±0.57	1.95±0.52	0.642
Global MFR, unitless	2.40±0.61	2.60±0.84	2.42±0.60	0.449
Minimal CVR, ml/min/g /mmHg	47.70±12.77	46.36±13.07	46.82±14.77	0.615
Rest LVOT gradient, mmHg	29±29	27±28	33±32	**<0.001**
Provoked LVOT gradient, mmHg	68±56	67±59	72±51	**<0.001**
LAD, cm	4.1±0.9	4.0±05	4.3±0.7	0.234
E/A ratio	1.7±1.0	1.4±0.6	1.4±0.5	0.382
E/e’ ratio	15.7±7.3	18.6 ±7.0	22.1±10.9	**0.009**
PASP, mmHg	34±10	34±8	37±10	0.456

Data are expressed as mean ± standard deviation.

MBF: myocardial blood flow; MFR: myocardial flow reserve; CVR: coronary vascular resistance; LVOT: left ventricular outflow tract; LAD: left atrial diameter; E/A:ratio of peak early diastolic velocity(E)/peak atrial filling velocity(A); E/e’: ratio of peak early diastolic velocity (E) /peak early diastolic velocity of the septal mitral annulus (e’); PASP: pulmonary artery systolic pressure

### Correlations

**Fig 3:**
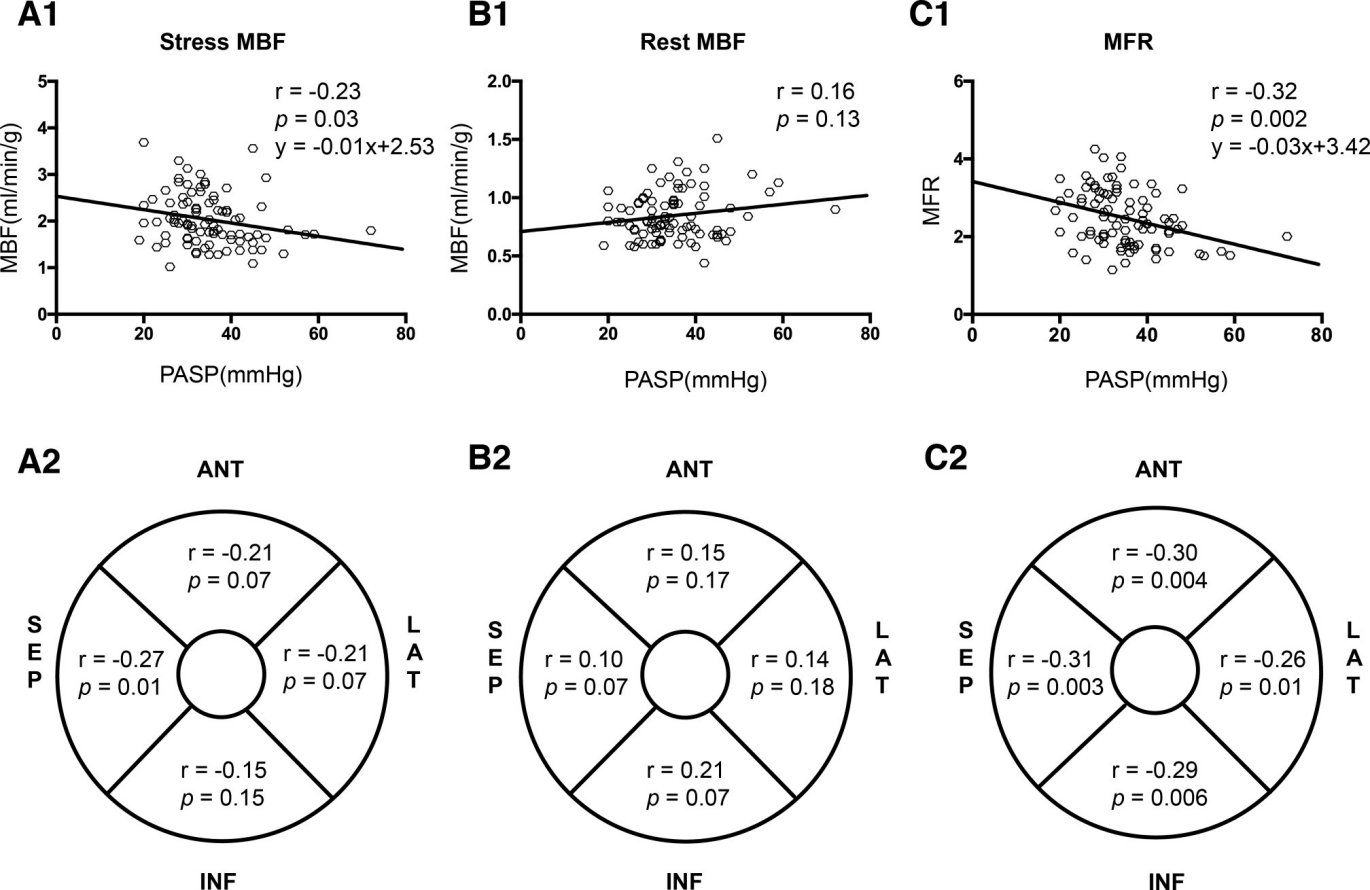
Correlation between PASP and PET parameters in the total HCM cohort. (A1,A2):correlation between PASP and global/regional stress MBF; (B1,B2): correlation between PASP and global/regional rest MBF; (C1,C2): correlation between PASP and global/regional MFR.

On the basis of findings that the echocardiographic parameters including PASP, LAD, E/A and E/e’ ratio were significantly different between patients with and without PH, we further investigate their correlations with the global MBF/MFR. The results showed that global stress MBF was negatively correlated with PASP values (r = -0.23; *p* < 0.05) and E/A ratio (r = -0.24; *p* < 0.05) but not LAD and E/e’ ratio; Similarly, global MFR was only negatively related to PASP values (r = -0.32; *p* < 0.01). On the other hand, the global MBFs at rest did not correlate with PASP (*p* > 0.05). Furthermore, the correlation between regional stress MBF/ MFR and PASP showed that the coefficients in septal wall were the highest (stress MBF: r = -0.27, p < 0.05; MFR: r = -0.32, *p* < 0.01, respectively) among all regional walls (Fig 3).

Univariate and multivariate regression analysis were further performed to identify the predictive factors for PH (all HCM patients). In univariable analysis, a significant correlation was found between PASP and global stress MBF, MFR, minimal CVR, LAD, E/A ratio and E/e′ ratio. On separate step-wise forward multiple linear regression, only global MFR(β = -0.35; *p* < 0.01), E/A ratio(β = 0.35; *p* < 0.01) and systolic blood pressure(β = 0.29; *p* < 0.01) were the independent predictors for PH as showed in Table 4.

**Table 4:** Regression: Correlation with the PASP.

	Univariable analysis		Multivariable analysis	
	β-coefficient	*p*-value	β-coefficient	*p*-value
Global stress MBF	-0.23	**0.030**		
Global MFR	-0.32	**0.002**	-0.35	**0.001**
Minimal CVR	-0.17	0.111		
SBP	0.14	0.213	0.29	**0.006**
DBP	0.13	0.216		
LAD	0.27	**0.011**		
E/A ratio	0.31	**0.003**	0.35	**0.001**
E/e’ ratio	0.41	**<0.001**		
Age	0.01	0.896		
BMI	-0.12	0.281		
Smoker	0.07	0.503		

PASP:pulmonary artery systolic pressures; MBF: myocardial blood flow;MFR: myocardial flow reserve; CVR: coronary vascular resistance; SBP: systolic blood pressure; DBP: diastolic blood pressure; LAD: left atrial diameter. BMI: body mass index; Multivariate regression model adjusted for minimal CVR,SBP,DBP, age, BMI and smoker, only independent variables that attained *p* < 0.05 are listed. E/A:ratio of peak early diastolic velocity(E)/peak atrial filling velocity(A); E/e’: ratio of peak early diastolic velocity (E) /peak early diastolic velocity of the septal mitral annulus (e’).

## Discussion

Our study demonstrates a link between echocardiography-estimated pulmonary pressure and the PET-measured MBF/MFR in HCM. These patients showed significantly more severe impairment of global and regional stress MBF and MFR than patients without PH. The study also indicates that global MFR which is independently determined for the change in PASP, indicating that impaired MFR is associated with PH in HCM patients.

Although invasive right heart catheterization remains the gold standard for assessment of pulmonary pressures, echocardiographic-derived measures of PASP was highly correlated to right heart catheterization in patients with left heart pathology [9, 17, 18], thereby validating echocardiography as a useful screen for PH. Estimating the exact prevalence of PH across echocardiographic PASP is definitely challenging because of the different thresholds used to define PH. In our cohort, using a cutoff at PASP>36 mmHg, PH was present in a significant proportion of our HCM population (36%), with moderate or severe in a small proportion (6%). The prevalence of PH was concordant with other left-side heart diseases such as aortic stenosis and heart failure with preserved ejection fraction which share similar haemodynamic features with HCM[3, 4].

In clinical practice, the impairment of diastolic function and left atrial dilation has been regarded as primary risk factors of PH in left-side heart disease[3]. Consistent with previous reports, our findings showed that the left atrium was significantly enlarged in PH-HCM patients compared with in no PH-HCM patients. Additionally, diastolic function, assessed by E/A and E/e’ ratio, was also significantly decreased in PH-HCM patients compared with in HCM patients. As in other left-heart diseases [3] or heart failure with preserved ejection fraction [19], PH appear to be the consequence of the increase of LV filling pressure, due to an impaired relaxation and augmented stiffness of the myocardium. HCM is a potentially inherited cardiomyopathy characterized by hypertrophy in the absence of another etiology. Some investigators have attributed diastolic dysfunction or mitral regurgitation to slow early ventricular filling associated with increased dependence on late diastolic filling by atrial contraction, thereby favoring the development of PH [20]. In the long-term, the PASP increase drives a progressive pulsatile loading of right ventricle, subsequently leading to right ventricle failure [21].

The concept of microvascular dysfunction as a precursor of HCM has been tested in multiple prior studies [7, 22]. It seems to be the result of structural changes in small vessels by luminal narrowing of the intramural microvascular network caused by hyperplasia and hypertrophy of the intima and media. Evidences have been showed that measurement of hyperemia MBF and MFR by means of PET is the most effective way to assess microvascular dysfunction in vivo [22]. A novel observation regards the role of microvascular dysfunction in PH in our HCM cohort. Global stress MBF and MFR values were generally depressed, whereas minimal CVR were elevated in PH-HCM patients compared with no PH-HCM patients. Based on previous studies [23, 24], MBF ≤ 1.8 mL/min/g or MFR ≤ 2.5 are used as a cutoff to distinguish abnormal or normal myocardial hyperemic flow increases, the number of patients with blunted stress MBF/MFR was much higher in PH-HCM patients. As intracoronary resistance relates inversely not only to the vessel diameter but also to the velocity of the blood flow [25]. Mechanistically, It was probably explained by the process that higher LV afterload and lower microvascular density in HCM patients may increase metabolic demand whereas reduce hyperemic flow, and thus myocardial perfusion, which in turn causes diastolic dysfunction resulting in increased left atrial pressure and thus pulmonary artery wedge pressure [26].

On the other hand, we found that the extent of global MFR were most negatively related to the severity of PASP, and the correlations were not improved in regional analysis, suggesting that the increased PASP in patients with HCM are not limited to microvascular dysfunction localized to certain walls but a diffuse process. In addition to traditional risk factors, including age, BMI, smoker, blood pressure, LA size and diastolic function, the global MFR was significantly associated with the PASP. Further multivariate linear regression analyses showed that global MFR, E/A ratio and SBP were independent predictive factors for PH in HCM patients. The progression of PH involves complex mechanisms including hemodynamic changes and cardiac function [27]. The potential for biomarker combinations is currently of considerable interest in the prediction of PH in HCM patients, and our study suggests that a combination of impaired global MFR, diastolic dysfunction and elevated blood pressure might be helpful for the screening and identification of PH in HCM patients.

In addition to MBF quantifications, we assessed LV function by means of gated PET and found the occurrence of abnormal LVEF reserve was in nearly a half of 89 HCM patients, consistent with two recent reports[16, 28]. Moreover, we showed a trend towards LVEF decrease after stress and blunted LVEF reserve, which appeared to be greater in those subjects with PH. The mechanism underling transient LV dysfunction has been partially elucidated by Dr. Gallagher [29] that vasodilator-induced redistribution of blood flow from the maximally vasodilated subendocardial layers to the subepicardial layers, resulting in ischemia. Since our study has excluded significant epicardial coronary stenosis, ischemia induced by microvascular dysfunction appears to play a role in the genesis of PH in HCM patients.

Of note, although LVOT obstruction is an established risk factor for adverse outcome in HCM patients, the effect of relief of LVOT obstruction after septal reduction therapy on PH was still paradoxical [4, 20]. Our data did not recognize an association between HCM phenotype (LVOT obstruction) and PASP or microvascular function. When taken in conjunction with our univariate and multivariate regression results, it appears that microvascular dysfunction could be a more powerful risk factor for PH-HCM.

There are some limitations of our study must be considered. Firstly, this was a retrospective study enrolled form a unique center. Secondly, assessment of pulmonary hemodynamics was derived from Doppler echocardiography but not the right heart catheterization, pulmonary pressure estimated from tricuspid regurgitation cannot distinguish whether the increase in pulmonary pressure is just a passive backward transmission of filling pressures driven by left ventricular diastolic dysfunction or secondary mitral regurgitation, atrial arrhythmias, or if there is also a superimposed component of pulmonary vasoconstriction due to progressive vascular remodeling, thereby limiting the accuracy of evaluation for PH. Finally, we are lacking complete data on other possible contributors to PH, such as RV function, RV MBF/MFR which could be of value for better understanding of pathophysiology in PH-HCM.

## Conclusion

Our study demonstrated that elevated PASP correlated with microvascular dysfunction in HCM patients. Global MFR was suggested to be an independent predictor for PH. Furthermore, global MFR, especially combined with diastolic dysfunction and elevated blood pressure demonstrated a better predictive value for PH-HCM. Our study may introduce a novel concept of a link between these two unfavorable disease features.

## Supporting information

S1 Table Estimated parameters from 17-segments myocardium kinetic modeling. Data are expressed as mean ± standard deviation.*p<0.05,* *p*<0.01, compared with stress parameters.F (ml/min/g): the transport rate constant from vascular space to myocardial tissue; k_2_ (min^-1^): the efflux rate constant from tissue to vascular space; k_3_ (min^-1^): the tracer metabolite rate constant trapped in tissue; V_b_ (ml/min/g): the fraction of blood volume in tissue.
(DOCX)

S2 Table Abnormal stress MBF and CFR between HCM patients with and without PH. Data are expressed as number of the patients(percentage). PH:pulmonary hypertension; MBF: myocardial blood flow; MFR: myocardial flow reserve.
(DOCX)

S3 Table PET-derived LVEF of HCM patients with and without PH. Data are expressed as number of the patients(percentage). PH: pulmonary hypertension; LVEF: left ventricular ejection fraction.
(DOCX)
