# Supplementary material for: Association of PET-measured myocardial flow reserve with echocardiography-estimated pulmonary artery systolic pressure in patients with hypertrophic cardiomyopathy
Source: PLoS One. 2019 Mar 20;14(3):e0212573. doi: 10.1371/journal.pone.0212573 (PMC6426216; doi:10.1371/journal.pone.0212573)
Supplement: S1 Table — Data are expressed as mean ± standard deviation.*p<0.05,* p<0.01, compared with stress parameters.F (ml/min/g): the transport rate constant from vascular space to myocardial tissue; k2 (min-1): the efflux rate constant from tissue to vascular space; k3 (min-1): the tracer metabolite rate constant trapped in tissue; Vb (ml/min/g): the fraction of blood volume in tissue. (DOCX) [file pone.0212573.s001.docx]

**S1 Table: Estimated parameters from 17-segments myocardium kinetic modeling**

|  | **Stress** | | | |  | **Rest** | | | |
| --- | --- | --- | --- | --- | --- | --- | --- | --- | --- |
| **Segment** | **F** | **k_2_** | **k_3_** | **V_b_** |  | **F** | **k_2_** | **k_3_** | **V_b_** |
| **Septal** |  |  |  |  |  |  |  |  |  |
| Apical_septal | 1.74±0.75 | 0.35±0.16 | 0.06±0.04 | 0.32±0.17 |  | 0.89±0.23** | 0.27±0.12** | 0.06±0.05 | 0.24±0.12** |
| Mid_anteroseptal | 2.00±0.79 | 0.33±0.14 | 0.05±0.03 | 0.30±0.16 |  | 0.85±0.23** | 0.17±0.08** | 0.05±0.05 | 0.21±0.09** |
| Mid_inferoseptal | 1.87±0.75 | 0.31±0.15 | 0.04±0.03 | 0.28±0.16 |  | 0.88±0.24** | 0.18±0.08** | 0.04±0.04 | 0.16±0.09** |
| Basal_anteroseptal | 1.78±0.55 | 0.31±0.17 | 0.06±0.04 | 0.46±0.16 |  | 0.84±0.22** | 0.23±0.13** | 0.07±0.06* | 0.32±0.12** |
| Basal_inferoseptal | 1.78±0.57 | 0.29±0.16 | 0.05±0.06 | 0.42±0.18 |  | 0.87±0.50** | 0.22±0.10** | 0.05±0.06 | 0.27±0.14** |
| **Lateral** |  |  |  |  |  |  |  |  |  |
| Apical_lateral | 2.13±0.74 | 0.40±0.18 | 0.05±0.03 | 0.26±0.17 |  | 0.82±0.22** | 0.22±0.10** | 0.04±0.03 | 0.09±0.08** |
| Mid_anterolateral | 2.36±0.96 | 0.37±0.19 | 0.05±0.03 | 0.39±0.22 |  | 0.88±0.24** | 0.21±0.10** | 0.04±0.03* | 0.10±0.08** |
| Mid_inferolateral | 2.39±0.85 | 0.41±0.19 | 0.05±0.03 | 0.31±0.18 |  | 0.82±0.23** | 0.21±0.10** | 0.04±0.03 | 0.11±0.06** |
| Basal_anterolateral | 2.70±1.05 | 0.37±0.22 | 0.05±0.05 | 0.40±0.21 |  | 0.86±0.26** | 0.23±0.12** | 0.05±0.03 | 0.15±0.07** |
| Basal_inferolateral | 2.42±0.81 | 0.46±0.23 | 0.05±0.04 | 0.36±0.17 |  | 0.78±0.22** | 0.240.10** | 0.04±0.03 | 0.14±0.06** |
| **Anterior** |  |  |  |  |  |  |  |  |  |
| Apical_anterior | 1.86±0.70 | 0.37±0.14 | 0.04±0.03 | 0.21±0.15 |  | 0.85±0.23** | 0.22±0.10** | 0.04±0.04 | 0.13±0.09** |
| Mid_anterior | 1.97±0.71 | 0.36±0.15 | 0.04±0.03 | 0.25±0.16 |  | 0.88±0.23** | 0.21±0.10** | 0.05±0.04 | 0.14±0.09** |
| Basal_anterior | 1.84±0.55 | 0.32±0.14 | 0.05±0.04 | 0.32±0.16 |  | 0.81±0.22** | 0.19±0.09** | 0.04±0.04 | 0.17±0.08** |
| **Inferior** |  |  |  |  |  |  |  |  |  |
| Apical_inferior | 1.86±0.87 | 0.38±0.17 | 0.05±0.04 | 0.29±0.19 |  | 0.84±0.25** | 0.23±0.10** | 0.05±0.04 | 0.16±0.11** |
| Mid_inferior | 1.97±0.72 | 0.36±0.14 | 0.04±0.03 | 0.25±0.16 |  | 0.82±0.24** | 0.21±0.14** | 0.04±0.04 | 0.09±0.07** |
| Basal_inferior | 2.10±0.58 | 0.40±0.16 | 0.04±0.03 | 0.33±0.15 |  | 0.78±0.23** | 0.22±0.10** | 0.05±0.04 | 0.15±0.09** |
| **Apex** | 1.77±0.72 | 0.41±0.18 | 0.04±0.03 | 0.24±0.14 |  | 0.90±0.26** | 0.27±0.12** | 0.05±0.04** | 0.19±0.13** |

Data are expressed as mean ± standard deviation.**p*<0.05,* *p*<0.01, compared with stress parameters.

F (ml/min/g): the transport rate constant from vascular space to myocardial tissue; k_2_ (min^-1^): the efflux rate constant from tissue to vascular space; k_3_ (min^-1^): the tracer metabolite rate constant trapped in tissue; V_b_ (ml/min/g): the fraction of blood volume in tissue.
