# Supplementary material for: Association of PET-measured myocardial flow reserve with echocardiography-estimated pulmonary artery systolic pressure in patients with hypertrophic cardiomyopathy
Source: PLoS One. 2019 Mar 20;14(3):e0212573. doi: 10.1371/journal.pone.0212573 (PMC6426216; doi:10.1371/journal.pone.0212573)
Supplement: S2 Table — Data are expressed as number of the patients(percentage). PH:pulmonary hypertension; MBF: myocardial blood flow; MFR: myocardial flow reserve. (DOCX) [file pone.0212573.s002.docx]

**S2 Table. Abnormal stress MBF and CFR between HCM patients with and without PH**

| **Characteristics** | **Total**  **(n=89)** | **No PH**  **(n=58)** | **PH**  **(n=31)** | ***p*-value** |
| --- | --- | --- | --- | --- |
| Stress MBF≤1.8 ml/min/g, n(%) | | | | |
| Global | 36(41) | 18(31) | 18(58) | **0.013** |
| Anterior | 49(55) | 27(46) | 22(71) | **0.027** |
| Septal | 45((50) | 25(43) | 20(64) | 0.054 |
| Lateral | 21(23) | 11(19) | 10(32) | 0.159 |
| Inferior | 43(48) | 25(43) | 18(58) | 0.178 |
| MFR≤2.5, n(%) | | | | |
| Global | 50(56) | 26(45) | 24(77) | **0.003** |
| Anterior | 56(63) | 30(52) | 26(84) | **0.003** |
| Septal | 42(47) | 23(40) | 19((61) | 0.051 |
| Lateral | 36(13) | 18(31) | 18(58) | **0.013** |
| Inferior | 47(53) | 26(45) | 21(68) | **0.039** |

Data are expressed as number of the patients(percentage). PH:pulmonary hypertension; MBF: myocardial blood flow; MFR: myocardial flow reserve.

.
