# Supplementary material for: Association of PET-measured myocardial flow reserve with echocardiography-estimated pulmonary artery systolic pressure in patients with hypertrophic cardiomyopathy
Source: PLoS One. 2019 Mar 20;14(3):e0212573. doi: 10.1371/journal.pone.0212573 (PMC6426216; doi:10.1371/journal.pone.0212573)
Supplement: S3 Table — Data are expressed as number of the patients(percentage). PH: pulmonary hypertension; LVEF: left ventricular ejection fraction. (DOCX) [file pone.0212573.s003.docx]

**S3 Table. PET-derived LVEF of HCM patients with and without PH**

| **Characteristics** | **Total**  **(n=89)** | **No PH**  **(n=58)** | **PH**  **(n=31)** | ***p*-value** |
| --- | --- | --- | --- | --- |
| Stress LVEF, % | 50±12 | 52±12 | 46±12 | **0.020** |
| Rest LVEF, % | 56±11 | 57±11 | 54±13 | 0.322 |
| LVEF reserve, % | -6±6 | -5±5 | -8±6 | **0.005** |
| Abnormal LVEF reserve, n(%) | 43(48) | 23(40) | 20(64) | **0.025** |

Data are expressed as number of the patients(percentage). PH: pulmonary hypertension; LVEF: left ventricular ejection fraction.
